# An early clinical trial of Salirasib, an oral RAS inhibitor, in Japanese patients with relapsed/refractory solid tumors

**DOI:** 10.1007/s00280-018-3618-4

**Published:** 2018-07-10

**Authors:** Junji Furuse, Takayasu Kurata, Naohiro Okano, Yasuhito Fujisaka, Daisuke Naruge, Toshio Shimizu, Hiroshi Kitamura, Tsutomu Iwasa, Fumio Nagashima, Kazuhiko Nakagawa

**Affiliations:** 1 Department of Medical Oncology, Kyorin University School of Medicine, 6-20-2 Shinkawa, Mitaka, Tokyo 181-8611 Japan; 2 Department of Medical Oncology, Kindai University Faculty of Medicine, 377-2 Ohno-Higashi, Osaka-Sayama, Osaka 589-8511 Japan

**Keywords:** *KRAS* mutation, Salirasib, *S*-*trans*, *Trans*-farnesylthiosalicylic acid, Phase I clinical trial, Relapsed/refractory solid tumors

## Abstract

**Purpose:**

Patients with *RAS*-positive tumors respond poorly to chemotherapies and have a few treatment options. Salirasib is an oral RAS inhibitor that competitively blocks the membrane association of RAS proteins. The aim of this phase I multiple-ascending-dose clinical trial was to investigate the safety and pharmacokinetics of Salirasib in Japanese patients with relapsed/refractory solid tumors and to explore its efficacy.

**Methods:**

Salirasib was started at a dose of 100-mg twice-daily and escalated to a maximum of 1000-mg twice-daily from days 1 to 21 of a 28-day regimen. The pharmacokinetics was evaluated on days 1 and 21. Dose-limiting toxicity (DLT) and adverse events (AEs) were monitored throughout the trial. Patients with stable disease or better repeated the dosing regimen.

**Results:**

A total of 21 patients received Salirasib. Among 14 patients tested, 4 had *KRAS* mutations. *C*_max_ and AUC_inf_ were maximal at 800 mg. No maximum tolerable dose was discerned, as no DLT was observed in any dosing group. The most frequently observed AEs were gastrointestinal disturbances, including diarrhea, abdominal pain, and nausea. No AEs led to discontinuation. All patients completed the first regimen and 11 patients repeated the regimen (median: 2 cycles; range: 1–13). Patients with *KRAS* mutations showed median progression-free survival of 227 days (range: 79–373).

**Conclusion:**

Salirasib was safe and well tolerated in Japanese patients, and 800-mg twice-daily is recommended for phase II trials. Although the number of participants with *KRAS* mutations was limited, the remarkably long progression-free period warrants further investigation.

**Clinical trial registration:**

JAPIC Clinical Trials Information; JapicCTI-121751.

**Electronic supplementary material:**

The online version of this article (10.1007/s00280-018-3618-4) contains supplementary material, which is available to authorized users.

## Introduction

Cancer is the leading cause of death in developed countries. Single or combination of chemotherapy, surgical resection, and radiation therapy is selected according to cancer type. Recent advances in cancer research and molecularly targeted drugs offer better chemotherapy options in various cancers. The RAS pathway is one of the most important pathways and has been the subject of molecularly targeted drugs [1].

RAS (three isoforms: KRAS, HRAS, and NRAS) is a key downstream effector of the epidermal growth factor (EGF) signaling pathway, regulating physiological cell proliferation, differentiation, and apoptosis [1]. The prevalence of *RAS* mutations is high in human cancers, being found in one-third of all cancers, 90% of pancreatic cancers, and 50% of colon cancers [1, 2]. Among the isoforms, *KRAS* is most commonly mutated [2]. In particular, because of the absence of symptoms, pancreatic cancer is often not found until advanced stage at which surgical resection may be not possible, resulting in a poor prognosis. Therefore, chemotherapy options to treat patients with *RAS* mutations are awaited.

Post-translationally farnesylated activated RAS binds to the plasma membrane and exerts a downstream signaling [3]. Mutated RAS is constitutively active without upstream EGF signaling, resulting in cancer-related uncontrolled RAS activities [1]. Several molecular drugs targeting EGF–RAS pathway have been developed, but their efficacy is not yet satisfactory in patients with *RAS* mutations [4, 5].

Salirasib is an S-trans, trans-farnesylthiosalicylic acid and a novel oral RAS inhibitor [6–8]. Salirasib mimics c-terminal farnesyl cysteine, common to all RAS isoforms, and competes with farnesylated RAS for putative-binding sites on the plasma membrane, leading to degradation of active cytoplasmic RAS [9, 10]. This mechanism of action may enable treatment of patients with *RAS* mutations who do not respond to the standard chemotherapies. The previous phase I/II trials in the USA (CCA-FTS-101A/B, 102–105, 201) showed good tolerability, but the efficacy in *RAS*-mutated patients was not conclusive [11, 12]. The present trial was a phase I trial to investigate the safety, tolerability, and pharmacokinetics of Salirasib in Japanese patients with relapsed/refractory solid tumors. The efficacy of Salirasib was also evaluated in an exploratory analysis.

## Materials and methods

### Patients and inclusion criteria

This trial was approved by the ethics committees of our institutions and was conducted in accordance with Declaration of Helsinki principles. Written informed consent was obtained from all participants.

Patients were male or female Japanese cancer patients aged ≥ 20 years at the time of obtaining written informed consent, who had relapsed/refractory solid tumors confirmed by histopathological examination, did not respond to standard therapies, and had expected survival of ≥ 3 months. Key inclusion criteria were creatinine ≤ 1.5 times upper limit of normal range (ULN); total bilirubin ≤ 2.0 mg/dL; aspartate aminotransferase (glutamate oxaloacetate transaminase) (AST [GOT]) and alanine aminotransferase (glutamate pyruvate transaminase) (ALT [GPT]) ≤ 3 times ULN; hemoglobin ≥ 9.0 g/dL; platelets ≥ 100 × 10^9^ cells/L; neutrophils ≥ 1.5 × 10^9^ cells/L. Patients with the following conditions were excluded: uncontrolled or severe concurrent medical conditions, including symptomatic primary/metastatic tumors in brain and meninges, renal/hepatic failures, and uncontrolled diabetes; complication of symptomatic ulceration or gastrointestinal conditions potentially interfering with oral administration and absorption; known infection with human immunodeficiency virus, hepatitis B, or hepatitis C; any chemotherapy and/or radiotherapy within 28 days of test drug administration.

Patients voluntarily participating in our *RAS* mutation study provided additional written informed consent. Formalin-fixed paraffin-embedded tumor samples from 14 patients were sectioned and stained with hematoxylin–eosin. Tumor regions were selected by pathologists at Kyorin University or Kinki University and DNA was extracted using the QIAamp DNA Micro kit (Qiagen, Hilden, Germany). Mutations in KRAS codons 12 and 13 were examined using a kit based on a Luminex assay (MEBGEN KRASMutation Detection kit, MBL, Nagoya, Japan).

### Study design

This trial was a multiple-ascending dose trial in 28-day cycles. The doses used were 100, 200, 400, 600, 800, and 1000 mg. The initial dose (100 mg) was determined in accordance with the International Conference on Harmonisation of Technical Requirements for Registration of Pharmaceuticals for Human Use-S9 guideline [13]. Salirasib was orally administered twice-daily after meals from days 1 to 21, followed by a 7-day pause. Patients received only the morning dose on day 1 for pharmacokinetics evaluation. Dose-limiting toxicity (DLT) was monitored throughout the 28-day regimen, with DLT defined as any of the following adverse events (AEs) for which a causal relationship with Salirasib was not ruled out: persistent grade 4 neutropenia for > 7 days; grade ≥ 3 febrile neutropenia; grade 4 thrombocytopenia or grade 3 thrombocytopenia requiring transfusion; nausea, vomiting, or diarrhea of grade ≥ 3 despite optimal treatment; grade ≥ 3 non-hematological toxicity leading to discontinuation of treatment. Grades were determined according to the National Cancer Institute Common Terminology Criteria for Adverse Events (NCI-CTCAE v.4.0).

Treatment was immediately terminated at the patient’s request when DLT occurred; the study physician decided that continuation was inappropriate because of AEs; or if the tumor was classified as progressive disease (PD) according to the Response Evaluation Criteria in Solid Tumor (RECIST) Guideline [14]. In cases of DLT, patients were allowed to continue the regimen with dose adjustments upon providing written agreement.

Patients were registered when up to three patients received ≥ 60% of planned treatment in the regimen before DLT was evaluated for dose escalation. If no DLT was observed in any patient, the dose was escalated to the next lowest level and new patients were registered until three patients received ≥ 60% of planned treatment in that dose group. If one patient showed DLT, patients were added to the group to give a final number of 6 patients with ≥ 60% of planned treatment, and it was confirmed that no other patient showed DLT before escalating to the next lowest dose. If ≥ 2 patients showed DLT in a dose group, the dose was to be considered to exceed the maximum tolerable dose and the ongoing regimen was to be discontinued. According to the DLT and pharmacokinetics data obtained by a given time, the recommended dose was determined and additional three patients were added to the dosing group a posteriori.

At the end of the regimen, patients were allowed to repeat the same regimen if all of the following criteria were met: no DLT occurred; the tumor was not determined as PD; the study physicians agreed; and the patient provided written informed consent. For the repeated regimen, Salirasib was administered twice-daily from day 1.

### Pharmacokinetics

Salirasib concentrations were measured in plasma and urine for pharmacokinetics analysis. Blood samples were collected 10 times on day 1 (before drug administration, and 0.5-, 1-, 1.5-, 2-, 4-, 6-, 8-, 12-, and 24-h post-administration), once on days 4, 7, and 14, and 9 times on day 21 (before morning dose, and 0.5, 1, 1.5, 2, 4, 6, 8, and 12 h after morning dose but before evening dose). Urine samples were collected from patients who received 200, 600, or 1000 mg on day 1 (before drug administration, and 0–6-, 6–12-, 12–24-h post-administration). Plasma and urine concentrations of Salirasib were determined by liquid chromatography–tandem mass spectrometry performed by LSI Medience Corporation (Tokyo, Japan).

### Safety

To evaluate the safety, vital signs (systolic and diastolic blood pressure, pulse rate, body temperature, and body weight), 12-lead electrocardiogram, clinical laboratory tests, chest X-ray, and performance status [Eastern Cooperative Oncology Group (ECOG)] were evaluated on predefined dates (Supplemental Table S1). Examined items in the clinical laboratory tests are listed in Supplemental Table S2.

AEs were graded in accordance with NCI-CTCAE v.4.0 and recorded. The study physicians determined whether the AEs were drug-related and recorded.

### Exploratory evaluation of efficacy

Diagnostic imaging using computed tomography, magnetic resonance imaging (MRI), fluorodeoxyglucose-positron emission tomography (PET), and/or bone scintigraphy was performed depending on the tumors and clinical symptoms on day 22 of first and repeated regimens or at discontinuation.

Specific tumor markers (e.g., carcinoembryonic antigen for colorectal cancer and prostate-specific antigen for prostate cancer) were measured on day 22 of first and repeated regimens or at discontinuation.

Based on the information obtained, the efficacy was scored in accordance with the RECIST guideline.

## Results

### Patients

A total of 23 patients were enrolled in the trial, and 21 patients received Salirasib treatment. The detailed patient characteristics are summarized in Table 1 and Supplemental Table S3.

**Table 1:** Patient demographics and baseline characteristics

	Total
Patients (*N*)	21
Sex, *N* (%)	
Male	14 (66.7)
Female	7 (33.3)
Age (years)	
< 65, *N* (%)	11 (52.4)
≥ 65, *N* (%)	10 (47.6)
Mean ± SD	62.5 ± 10.6
Median	63.0
Range, 52–74	43–80
ECOG performance status, *N* (%)	
0	16 (76.2)
1	5 (23.8)
2	0 (0.0)
Tumor type, *N* (%)	
Lung	0 (0.0)
Pancreas	5 (23.8)
Colorectal	7 (33.3)
Stomach	0 (0.0)
Esophagus	1 (4.8)
Biliary tract	4 (19.0)
Liver	0 (0.0)
Others	4 (19.0)
Stage of cancer, *N* (%)	
IV	9 (42.9)
IVB	2 (9.5)
Refractory	10 (47.6)
Histopathological classification, *N* (%)	
Small round cell tumor	1 (4.8)
Invasive ductal carcinoma	1 (4.8)
Adenocarcinoma	15 (71.4)
Clear cell carcinoma	1 (4.8)
Squamous cell carcinoma	1 (4.8)
Acral lentiginous melanoma	1 (4.8)
Unknown	1 (4.8)
TNM staging (T) at study onset, *N* (%)	
T0	15 (71.4)
T3	3 (14.3)
T4	3 (14.3)
TNM staging (N) at study onset, *N* (%)	
N0	11 (52.4)
N1	6 (28.6)
N2	1 (4.8)
N2b	2 (9.5)
N3	1 (4.8)
TNM staging (M) at study onset, *N* (%)	
M0	1 (4.8)
M1	15 (71.4)
M1b	4 (19.0)
M1c	1 (4.8)
Treatment history: surgery^a^, *N* (%)	
0	5 (23.8)
≥ 1	16 (76.2)
Complete resection^a^	10 (47.6)
Residual tumor present^a^	7 (33.3)
Unknown^a^	3 (14.3)
Treatment history: radiotherapy, *N* (%)	
0	18 (85.7)
≥ 1	3 (14.3)
Prior systemic regimens, *N* (%)	
0	0 (0.0)
1	3 (14.3)
2	6 (28.6)
3	3 (14.3)
4	3 (14.3)
5	2 (9.5)
6	4 (19.0)
*KRAS* mutations determined, *N* (%)	4 (19.0)

*ECOG* Eastern Cooperative Oncology Group

^a^Multiple answers were counted cumulatively

### Pharmacokinetics

**Fig. 1:**
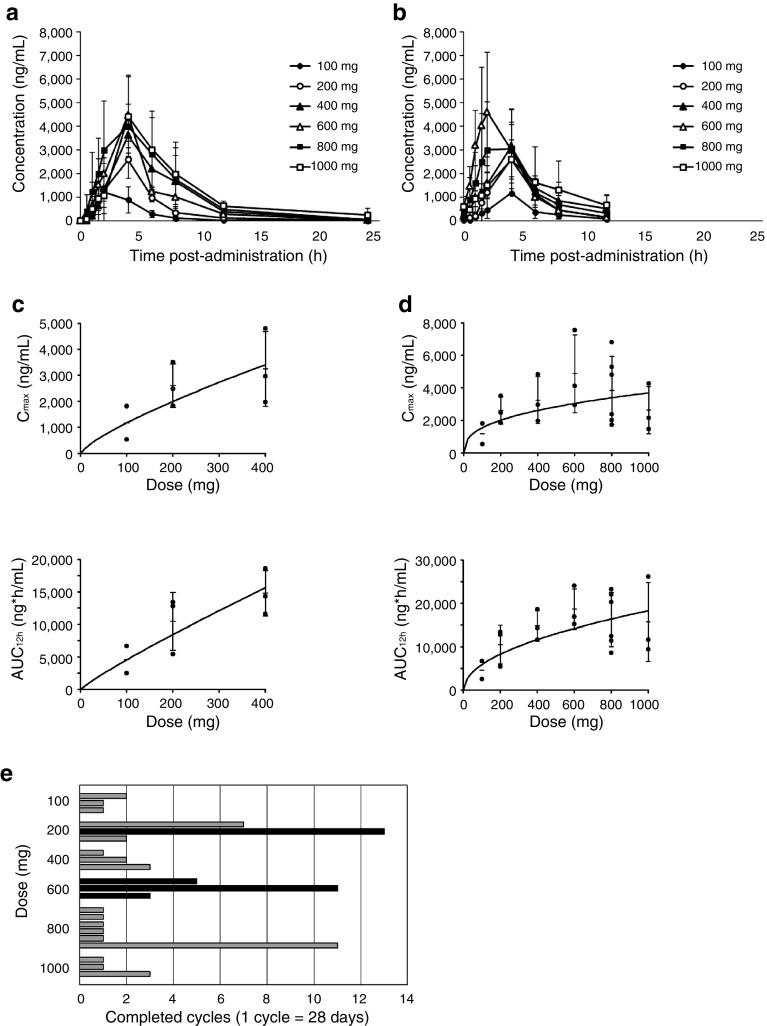
**a** Changes in plasma concentrations of Salirasib over 24 h after single administration (day 1 of the first cycle). At median of 1.97–4.00-h post-administration, plasma Salirasib levels reached mean *C*_max_ of 1340–4990 ng/mlL. **b** Changes in plasma concentration of Salirasib over 24 h after multiple administrations (day 21 of the first cycle). At median of 2.10–4.05-h post-administration, plasma Salirasib levels reached mean *C*_max_ of 1180–4870 ng/mL. There were no apparent differences between pharmacokinetic parameters after single compared to multiple administrations. **c** Power model analyses for *C*_max_ (top) and AUC_12h_ (bottom) for 0–400-mg dosing on day 21 of the first cycle. Up to 400 mg, *C*_max_ and AUC_12h_ increased proportionally and in dose-dependent manners. **d** Power model analyses for *C*_max_ (top) and AUC_12h_ (bottom) for 0–1000-mg dosing on day 21 of the first cycle. Plasma concentration of Salirasib did not proportionally increase after administration of 600 mg and higher doses. **e** Numbers of cycles completed. Patients who received ≥ 60% of planned medication before discontinuation were considered to have completed the regimen. All 21 patients completed at least the first regimen. Note the successful repeated regimens over a long period without progressive disease in patients with known *KRAS* mutations (black bars). AUC_12h_ area under the plasma concentration–time curve from time 0 to 12 h, *C*_max_ maximum plasma concentration

Pharmacokinetics was analyzed in terms of plasma concentrations. Changes in plasma Salirasib levels over 24 h on days 1 and 21 are displayed in Fig. 1a, b. Plasma Salirasib reached maximum plasma concentration (*C*_max_) at median of 1.97–4.00 h and half-life (*t*_1/2_) at mean of 3.5–9.11-h post-administration on day 1, and *C*_max_ at 2.10–4.05 h on day 21. There was no apparent difference in pharmacokinetic parameters after single administration (day 1) compared to multiple administrations (day 21) (Table 2).

**Table 2:** Pharmacokinetic parameters of Salirasib in the first regimen (days 1 and 21)

	100 mg	200 mg	400 mg	600 mg	800 mg	1000 mg
Pharmacokinetic parameters on day 1						
Patients (*N*)	3	3	3	3	6	3
*C*_max_ (ng/mL), mean ± SD	1340 ± 473	2830 ± 822	3630 ± 876	4500 ± 1580	4790 ± 1100	4990 ± 1210
*t*_max_ (h)						
Median	1.97	3.98	4.00	3.98	3.99	3.97
Range	1.52–3.88	2.13–4.00	4.00–4.03	1.42–4.00	2.02–5.93	3.97–6.37
AUC_inf_ (ng h/mL), mean ± SD	4970 ± 1600	11,400 ± 3580	20,300 ± 3710	18,500 ± 3060	26,600 ± 4200	26,900^a^
AUC_12h_ (ng h/mL), mean ± SD	4700 ± 1490	10,400 ± 3180	18,000 ± 4060	16,900 ± 2880	23,800 ± 3010	22,400 ± 2890
*t*_1/2_ (h), mean ± SD	3.81 ± 0.25	4.16 ± 1.13	4.00 ± 1.88	3.50 ± 0.39	3.67 ± 0.81	9.11 ± 9.79
CL/F (L/h), mean ± SD	21.6 ± 6.80	19.1 ± 7.35	20.1 ± 3.83	33.0 ± 5.25	30.7 ± 4.55	38.3^a^
Pharmacokinetic parameters on day 21						
Patients (*N*)	2	3	3	3	6	3
*C*_max_ (ng/mL), mean ± SD	1180^a1^	2610 ± 837	3250 ± 1440	4870 ± 2390	3840 ± 2090	2630 ± 1450
*t*_max_ (h)						
Median	4.05	3.93	3.97	2.10	2.98	3.95
Range	4.05–4.05	3.92–4.00	3.97–6.00	2.00–4.00	1.42–5.98	2.00–3.97
AUC_12h_ (ng h/mL), mean ± SD	4610^a^	10,500 ± 4450	14,800 ± 3520	18,700 ± 4710	16,300 ± 6240	15,700 ± 9100

*AUC*_*12h*_ area under the plasma concentration–time curve from time 0–12 h, *AUC*_*inf*_ area under the plasma concentration–time curve from time 0 to infinity, CL/F apparent clearance, *C*_*max*_ maximum plasma concentration, *t*_*1*/*2*_ half-life, *t*_*max*_ time of maximum plasma concentration

^a^SD was not calculated because of missing data

Power model analyses revealed that *C*_max_ and area under the plasma concentration–time curve from time 0 to infinity (AUC_inf_) increased proportionally until 400 mg in dose-dependent manners (Fig. 1c, d), and less than proportionally until 800 mg, with no difference between 800- and 1000-mg groups (Table 2).

Within 24 h, Salirasib was not excreted in urine in any dosing group.

### Safety

During the treatment, no DLT was observed and all patients completed the first regimen. Therefore, all dosing regimens (100-, 200-, 400-, 600-, 800-, and 1000-mg twice-daily) were administered with three patients in each group, and three additional patients were added a posteriori in the 800 mg group.

In the first and repeated regimens, AEs were observed in all 21 patients, with drug-related AEs in all patients except two (one patient each in 100- and 1000-mg groups). Occurrences of AEs are summarized in Table 3.

**Table 3:** Summary of occurrences of adverse events (AEs) in the first and repeated regimens

	100 mg	200 mg	400 mg	600 mg	800 mg	1000 mg	Total
Patients (*N*)	3	3	3	3	6	3	21
All AEs, *N* (%)	3 (100)	3 (100)	3 (100)	3 (100)	6 (100)	3 (100)	21 (100)
Patients with AEs of grade ≥ 3, *N* (%)	2 (66.7)	1 (33.3)	2 (66.7)	0 (0.0)	2 (33.3)	2 (66.7)	9 (42.9)
Serious AEs, *N* (%)	1 (33.3)	0 (0.0)	0 (0.0)	0 (0.0)	0 (0.0)	0 (0.0)	1 (4.8)
Discontinuation because of AEs, *N* (%)	0 (0.0)	0 (0.0)	0 (0.0)	0 (0.0)	0 (0.0)	0 (0.0)	0 (0.0)
All drug-related AEs, *N* (%)	2 (66.7)	3 (100)	3 (100)	3 (100)	6 (100)	2 (66.7)	19 (90.5)
Patients with drug-related AEs of grade ≥ 3, *N* (%)	0 (0.0)	0 (0.0)	1 (33.3)	0 (0.0)	1 (16.7)	2 (66.7)	4 (19.0)
Serious drug-related AEs, *N* (%)	0 (0.0)	0 (0.0)	0 (0.0)	0 (0.0)	0 (0.0)	0 (0.0)	0 (0.0)
Discontinuation because of drug-related AEs, *N* (%)	0 (0.0)	0 (0.0)	0 (0.0)	0 (0.0)	0 (0.0)	0 (0.0)	0 (0.0)
Dose-limiting toxicity (DLT), *N* (%)	0 (0.0)	0 (0.0)	0 (0.0)	0 (0.0)	0 (0.0)	0 (0.0)	0 (0.0)
Death, *N* (%)	0 (0.0)	0 (0.0)	0 (0.0)	0 (0.0)	0 (0.0)	0 (0.0)	0 (0.0)
AEs observed in ≥ 3 patients, *N* (%)							
Diarrhea	2 (66.7)	2 (66.7)	3 (100)	3 (100)	6 (100)	2 (66.7)	18 (85.7)
Abdominal pain	1 (33.3)	0 (0.0)	3 (100)	0 (0.0)	1 (16.7)	1 (33.3)	6 (28.6)
Nausea	1 (33.3)	0 (0.0)	2 (66.7)	2 (66.7)	1 (16.7)	0 (0.0)	6 (28.6)
Decreased appetite	1 (33.3)	0 (0.0)	1 (33.3)	1 (33.3)	4 (66.7)	2 (66.7)	9 (42.9)
Vomiting	0 (0.0)	0 (0.0)	1 (33.3)	2 (66.7)	1 (16.7)	0 (0.0)	4 (19.0)
AEs observed at grade ≥ 3, *N* (%)							
Diarrhea	0 (0.0)	0 (0.0)	0 (0.0)	0 (0.0)	1 (16.7)	1 (33.3)	2 (9.5)
Anemia	1 (33.3)	0 (0.0)	0 (0.0)	0 (0.0)	0 (0.0)	1 (33.3)	2 (9.5)
Ascites	1 (33.3)	0 (0.0)	0 (0.0)	0 (0.0)	0 (0.0)	0 (0.0)	1 (4.8)
Cholestatic jaundice	0 (0.0)	1 (33.3)	0 (0.0)	0 (0.0)	0 (0.0)	0 (0.0)	1 (4.8)
Amylase increased	0 (0.0)	0 (0.0)	1 (33.3)	0 (0.0)	0 (0.0)	0 (0.0)	1 (4.8)
Hyponatremia	1 (33.3)^a^	0 (0.0)	1 (33.3)	0 (0.0)	0 (0.0)	0 (0.0)	2 (9.5)
Hypercalcemia	0 (0.0)	0 (0.0)	1 (33.3)	0 (0.0)	0 (0.0)	0 (0.0)	1 (4.8)
Hypophosphatemia	0 (0.0)	0 (0.0)	1 (33.3)	0 (0.0)	0 (0.0)	0 (0.0)	1 (4.8)
Bilirubin increased	0 (0.0)	1 (33.3)	0 (0.0)	0 (0.0)	0 (0.0)	0 (0.0)	1 (4.8)
γ-GTP increased	1 (33.3)	1 (33.3)	0 (0.0)	0 (0.0)	0 (0.0)	1 (33.3)	3 (14.3)
AST increased	0 (0.0)	1 (33.3)	0 (0.0)	0 (0.0)	0 (0.0)	0 (0.0)	1 (4.8)
ALP increased	0 (0.0)	1 (33.3)	0 (0.0)	0 (0.0)	1 (16.7)	1 (33.3)	3 (14.3)
Troponin T increased	0 (0.0)	0 (0.0)	0 (0.0)	0 (0.0)	0 (0.0)	1 (33.3)	1 (4.8)
Hemoglobin decreased	1 (33.3)	0 (0.0)	0 (0.0)	0 (0.0)	0 (0.0)	1 (33.3)	2 (9.5)
Lymphopenia	1 (33.3)^a^	0 (0.0)	0 (0.0)	0 (0.0)	0 (0.0)	0 (0.0)	1 (4.8)
Decreased appetite	1 (33.3)	0 (0.0)	0 (0.0)	0 (0.0)	0 (0.0)	0 (0.0)	1 (4.8)
Cancer pain	1 (33.3)	0 (0.0)	0 (0.0)	0 (0.0)	0 (0.0)	0 (0.0)	1 (4.8)

*ALP* alkaline phosphatase, *AST* aspartate aminotransferase, *γ-GTP* gamma-glutamyl transpeptidase

^a^Grade 4

Most frequent AEs observed were gastrointestinal disturbances, occurring in more than two-thirds of patients in every dosing group. Drug-related AEs observed in ≥ 3 patients were diarrhea, abdominal pain, nausea, decreased appetite, and vomiting (Table 3). AEs of grade ≥ 3 were not observed in the first or repeated regimens in the 600-mg group, but occasionally in all other dosing groups (Table 3). One serious AE (ascites) was found during the first regimen in the 100-mg group, but was not considered test drug-related. Otherwise, no serious AEs were observed in any dose group, and no AEs led to the early discontinuation of the regimen. No patients died during the treatment period.

### Exploratory evaluation of efficacy

An exploratory efficacy evaluation of Salirasib was carried out. Response rates are listed in Table 4. Neither complete response (CR) nor partial response (PR) was found during the trial. Best response rates observed were stable disease (SD) (0/3, 2/3, 1/3, 2/3, 1/6, and 1/3 patients in 100, 200, 400, 600, 800, and 1000 mg groups, respectively). All patients completed the first regimen and 11 patients repeated the regimen (Fig. 1e). Median overall total number of repeated cycles was 2 (range: 1–13) and median overall progression-free survival was 53 days (range: 16–373) (Table 4).

**Table 4:** Efficacy analyses assessed by physicians at the study sites

	100 mg	200 mg	400 mg	600 mg	800 mg	1000 mg	Total
Patients (*N*)	3	3	3	3	6	3	21
Response rate^a^, *N* (%)							
Complete response (CR)	0 (0.0)	0 (0.0)	0 (0.0)	0 (0.0)	0 (0.0)	0 (0.0)	0 (0.0)
Partial response (PR)	0 (0.0)	0 (0.0)	0 (0.0)	0 (0.0)	0 (0.0)	0 (0.0)	0 (0.0)
Stable disease (SD)	1 (33.3)	3 (100.0)	2 (66.7)	2 (66.7)	1 (16.7)	1 (33.3)	10 (47.6)
Progressive disease (PD)	2 (66.7)	0 (0.0)	1 (33.3)	1 (33.3)	3 (50.0)	2 (66.7)	9 (42.9)
Objective response rate (ORR): CR + PR	0 (0.0)	0 (0.0)	0 (0.0)	0 (0.0)	0 (0.0)	0 (0.0)	0 (0.0)
Disease control rate (DCR): CR + PR + SD	1 (33.3)	3 (100.0)	2 (66.7)	2 (66.7)	1 (16.7)	1 (33.3)	10 (47.6)
Number of cycles completed^b^							
Median	1	7	2	5	1	1	2
Range	1–2	2–13	1–3	3–11	1–11	1–3	1–13
Progression-free survival^c^ (days)							
Median	22	213	56	135	29	22	53
Range	16–53	57–373	22–91	79–319	22–307	22–91	16–373

^a^Two patients in 800-mg group were not evaluated

^b^It was recorded as “completed” when patients underwent ≥ 60% of planned treatment in the ongoing regimen

^c^For one patient in 800-mg group, days until withdrawal were counted, because of the early discontinuation but without PD

All four patients with *KRAS* mutations repeated the regimen (median: 8 cycles; range: 3–13) (Fig. 1e). Median progression-free survival in patients with *KRAS* mutations was 227 days (range: 79–373).

## Discussion

This phase I clinical trial investigated the safety and tolerability of Salirasib, a novel oral RAS inhibitor, for the first time in Japanese patients.

Throughout the trial, including first and repeated regimens, frequency and severity of AEs and drug-related AEs did not apparently differ in any dose groups. Most frequently observed drug-related AEs were gastrointestinal toxicities, such as diarrhea, abdominal pain, and nausea, consistent with the previous phase I/II trials carried out in US patients [11, 12, 15]. In all dose groups, no DLT was observed and no patients discontinued the trial for AEs. Therefore, Salirasib is considered to be safe and tolerable up to 1000 mg in Japanese patients. In a phase I trial in the US patients [15], no DLT was observed up to 800 mg, although all patients on 800-mg regimen experienced drug-related AEs, consistent with our trial. Pharmacokinetic analyses in our study showed that C_max_ and AUC increased with escalating doses of up to 800 mg. These results together with the safety profile indicate that 800 mg twice-daily is the recommended dose for Japanese patients. The tolerability was demonstrated across all types of cancers investigated in the study. Therefore, no cancer types have to be excluded from future trials in terms of safety.

In a previous phase II trial in patients with *KRAS* mutations, no patients achieved PR [11]. Consistent with the study, no patients showed CR or PR with Salirasib in our trial. Although the efficacy data are not conclusive due to the small number of patients included, it is worth noting the long period of SD and number of repeated treatments in the present trial. Therefore, further study to investigate the efficacy of Salirasib is encouraged.

Patients with *KRAS*-mutated tumors respond to chemotherapies poorly. EGF receptor inhibitors (e.g., panitumumab) show a little efficacy in patients with *KRAS* mutations [4, 16, 17], owing to EGF receptor-independent constitutive activities of RAS mutants. Molecular drugs targeting prenylation of RAS, such as farnesyl transferase inhibitors (e.g., tipifarnib), also showed a little activity in clinical trials [18–20], partially because membrane association of KRAS and NRAS mutants can still take place via alternative modification pathway through geranylgeranyltransferase-I, even though farnesylation of RAS is successfully inhibited [21, 22]. In turn, although no patients achieved PR, all patients with *KRAS* mutations in this study repeated the Salirasib regimen before they were discontinued due to PD, demonstrating its potential to control disease activity. To our knowledge, this is the first indication that Salirasib may be effective treatment for patients with *KRAS* mutations who are non-responsive to other chemotherapies.

In future trials, it would also be of interest to compare the efficacy in cohorts stratified according to the presence of *RAS* mutations. Efficacy analyses could be performed in patients with pancreatic or colorectal cancers, because *RAS* mutations are more frequent in those types of cancers. Furthermore, in lung adenocarcinoma, it has been demonstrated that *KRAS* mutation induces the expression of programmed death ligand 1 (PD-L1) [23, 24]. Accordingly, combination therapy of Salirasib with an anti-PD-1 agent may enhance the anti-cancer efficacy.

Salirasib was well tolerated, and although our efficacy analyses were not conclusive, the results indicate that RAS inhibitors are a promising molecular approach, especially for patients with *KRAS* mutations who currently have no other effective therapy options.

## Electronic supplementary material

Below is the link to the electronic supplementary material.

Supplementary material 1 (PDF 64 KB)
