# Supplementary material for: An early clinical trial of Salirasib, an oral RAS inhibitor, in Japanese patients with relapsed/refractory solid tumors
Source: Cancer Chemother Pharmacol. 2018 Jul 10;82(3):511–9. doi: 10.1007/s00280-018-3618-4 (PMC6105164; doi:10.1007/s00280-018-3618-4)
Supplement: Supplementary file 1 — Supplementary material 1 (PDF 64 KB) [file 280_2018_3618_MOESM1_ESM.pdf]

Supplementary material (online only)

Article title An early clinical trial of Salirasib, an oral Ras inhibitor, in Japanese patients with relapsed/refractory solid tumors

Journal name Cancer Chemotherapy and Pharmacology

Author names Junji Furuse, Takayasu Kurata, Naohiro Okano, Yasuhito Fujisaka, Daisuke Naruge, Toshio Shimizu, Hiroshi Kitamura,Tsutomu Iwasa, Fumio Nagashima and Kazuhiko Nakagawa

Affiliation and e-mail address of the corresponding author

Junji Furuse, Department of Medical Oncology Kyorin University School of Medicine, jfuruse@ks.kyorin-u.ac.jp.

Supplementary Table S1

Safety evaluation schedule

	Screening	First regimen									Repeated regimen				At discontinuation
Day	−14 to −1	1*[1]	1*[2]	2*[1]	4*[1]	7*[1]	14*[1]	21	22	28	1	8	22	28	
Written informed consent	X*[3]										X*[4]				
Body weight	X					X			X	X		X	X	X	X
Systolic and diastolic blood pressure		X	X*[5]	X	X	X	X		X	X		X	X	X	X
Pulse rate		X	X*[5]	X	X	X	X		X	X		X	X	X	X
Body temperature		X	X*[5]	X	X	X	X		X	X		X	X	X	X

	Screening	First regimen									Repeated regimen				At discontinuation
Day	−14 to −1	1[*1]	1[*2]	2[*1]	4[*1]	7[*1]	14[*1]	21	22	28	1	8	22	28	
12-lead electrocardiogram	X		X[*6]	X	X	X	X		X	X		X	X	X	X
Pregnancy test	X									X				X	X
ECOG performance status	X					X	X		X	X		X	X	X	X
General clinical test	X			X		X	X		X	X		X[*7]	X	X	X
Tumor evaluation by CT, MRI, and tumor marker test	X								X				X[*8]		X
Chest X-ray	X								X				X		X
Tumor biopsy	(X)														
Blood sampling for pharmacokinetic evaluation		X[*9]	X[*10]	X	X	X	X	X[*11]							
Urine sampling for pharmacokinetic evaluation[12]		X	X												
Gene expression analyses	X			X					X	X			X	X	X
AE monitoring		Continuous													

X: Required item; (X): Optional item (examined only with written agreement provided by patients).

[*1]Pre-administration (morning dose).

[*2]Post-administration.

[*3]Test drug administration was commenced within 30 days after patients provided written informed consent.

[*4]Only patients who provided written consent after completing the first regimen entered the repeated-dose period.

[*5]Measurements were carried out at 0.5, 1, 1.5, 2, 4, 6, and 8 hours after drug administration.

[*6]Measurements were carried out at 1, 2, 4, 6, and 8 hours after administration.

[*7]No qualitative urine analyses.

[*8]CT or MRI imaging was obligated to be carried out once every two regimens (in odd-number regimens).

[*9]With written agreement, blood samples for genotyping were also collected.

[*10]Blood samples were collected at 0.5, 1, 1.5, 2, 4, 6, 8, and 12 hours after drug administration.

[*11]Blood samples were collected before administration (morning), and at 0.5, 1, 1.5, 2, 4, 6, 8, and 12 hours after administration (before evening administration).

[*12]Urine samples were collected from patients who received 200, 600, and 1000 mg of salirasib on day 1 (before drug administration; 0–6, 6–12, 12–24 hours post-administration).

Supplementary Table S2

Items examined in the clinical laboratory tests

Clinical test	Items
Blood chemical analyses	Na, K, Cl, Ca, P, Mg, BUN, creatinine, blood glucose, total protein, albumin, total bilirubin, γ-GTP, AST (GOT), ALT (GPT), ALP, LDH, total cholesterol, triglyceride, cardiac troponin T
Hematologic tests	Hemoglobin, hematocrit, RBC count, WBC count, lymph count, monocyte count, neutrophil count, eosinophil count, basophil count, platelet count
Urine analyses	Protein, glucose, urobilinogen, pH, ketone bodies, occult blood

Supplementary Table S3

Summary statistics of patient characteristics

	100 mg	200 mg	400 mg	600 mg	800 mg	1000 mg	Total
Patients (*N*)	3	3	3	3	6	3	21
Sex, *N* (%)							
Male	1 (33.3)	2 (66.7)	3 (100.0)	1 (33.3)	4 (66.7)	3 (100.0)	14 (66.7)
Female	2 (66.7)	1 (33.3)	0 (0.0)	2 (66.7)	2 (33.3)	0 (0.0)	7 (33.3)
Age (years)							
<65, *N* (%)	1 (33.3)	2 (66.7)	1 (33.3)	1 (33.3)	5 (83.3)	1 (33.3)	11 (52.4)
≥65, *N* (%)	2 (66.7)	1 (33.3)	2 (66.7)	2 (66.7)	1 (16.7)	2 (66.7)	10 (47.6)
Mean ± SD	66.0 ± 12.2	64.0 ± 7.8	64.3 ± 16.0	61.3 ± 15.9	60.0 ± 11.3	61.7 ± 5.8	62.5 ± 10.6
Median	72.0	60.0	65.0	70.0	63.0	65.0	63.0
Range	52–74	59–73	48–80	43–71	43–76	55–65	43–80
Height (cm)							
Mean ± SD	166.20 ± 7.76	161.27 ± 6.01	162.80 ± 11.09	160.47 ± 7.20	160.98 ± 11.01	171.80 ± 6.95	163.50 ± 8.82
Median	166.70	158.10	161.20	162.40	161.55	171.50	163.80
Range	158.2–173.7	157.5–168.2	152.6–174.6	152.5–166.5	147.6–176.9	165.0–178.9	147.6–178.9
Weight (kg)							
Mean ± SD	62.20 ± 3.82	62.07 ± 9.38	50.47 ± 9.81	52.23 ± 6.79	59.72 ± 7.79	66.83 ± 15.71	59.03 ± 9.72
Median	61.80	64.20	48.80	54.50	59.00	66.10	58.80
Range	58.6–66.2	51.8–70.2	41.6–61.0	44.6–57.6	51.1–71.2	51.5–82.9	41.6–82.9

	100 mg	200 mg	400 mg	600 mg	800 mg	1000 mg	Total
Patients (*N*)	3	3	3	3	6	3	21
Body surface area (m^2)							
Mean ± SD	1.692 ± 0.058	1.653 ± 0.146	1.525 ± 0.201	1.529 ± 0.079	1.626 ± 0.167	1.783 ± 0.232	1.634 ± 0.164
Median	1.704	1.655	1.494	1.540	1.637	1.777	1.629
Range	1.63–1.74	1.51–1.80	1.34–1.74	1.45–1.60	1.44–1.88	1.55–2.02	1.34–2.02
BMI (kg/m^2)							
Mean ± SD	22.65 ± 2.83	23.79 ± 2.56	18.88 ± 1.08	20.45 ± 3.99	23.00 ± 1.23	22.43 ± 3.49	22.03 ± 2.73
Median	23.82	24.81	18.78	19.66	22.96	22.47	22.62
Range	19.4–24.7	20.9–25.7	17.9–20.0	16.9–24.8	21.0–24.6	18.9–25.9	16.9–25.9
Time from primary diagnosis (days)							
Mean ± SD	656.3 ± 188.3	800.0 ± 601.1	819.3 ± 421.4	1161.7 ± 610.7	1016.2 ± 339.2	727.3 ± 456.1	885.3 ± 417.4
Median	700.0	1137.0	939.0	1249.0	927.5	870.0	870.0
Range	450–819	106–1157	351–1168	512–1724	667–1529	217–1095	106–1724
ECOG performance status, *N* (%)							
0	3 (100.0)	3 (100.0)	2 (66.7)	2 (66.7)	5 (83.3)	1 (33.3)	16 (76.2)
1	0 (0.0)	0 (0.0)	1 (33.3)	1 (33.3)	1 (16.7)	2 (66.7)	5 (23.8)
2	0 (0.0)	0 (0.0)	0 (0.0)	0 (0.0)	0 (0.0)	0 (0.0)	0 (0.0)
Smoking (number of cigarettes per day), *N* (%)							
None	3 (100.0)	3 (100.0)	2 (66.7)	3 (100.0)	5 (83.3)	2 (66.7)	18 (85.7)
<5	0 (0.0)	0 (0.0)	0 (0.0)	0 (0.0)	0 (0.0)	0 (0.0)	0 (0.0)
≥5–<20	0 (0.0)	0 (0.0)	1 (33.3)	0 (0.0)	1 (16.7)	1 (33.3)	3 (14.3)
≥20	0 (0.0)	0 (0.0)	0 (0.0)	0 (0.0)	0 (0.0)	0 (0.0)	0 (0.0)

	100 mg	200 mg	400 mg	600 mg	800 mg	1000 mg	Total
Patients (*N*)	3	3	3	3	6	3	21
Alcohol consumption frequency, *N* (%)							
None	3 (100.0)	1 (33.3)	1 (33.3)	3 (100.0)	4 (66.7)	2 (66.7)	14 (66.7)
3–4 times/ month	0 (0.0)	2 (66.7)	1 (33.3)	0 (0.0)	0 (0.0)	0 (0.0)	3 (14.3)
3–4 times/ week	0 (0.0)	0 (0.0)	0 (0.0)	0 (0.0)	0 (0.0)	0 (0.0)	0 (0.0)
Once/ day	0 (0.0)	0 (0.0)	1 (33.3)	0 (0.0)	2 (33.3)	1 (33.3)	4 (19.0)
Tumor type, *N* (%)							
Lung	0 (0.0)	0 (0.0)	0 (0.0)	0 (0.0)	0 (0.0)	0 (0.0)	0 (0.0)
Pancreas	2 (66.7)	0 (0.0)	1 (33.3)	1 (33.3)	0 (0.0)	1 (33.3)	5 (23.8)
Colorectal	0 (0.0)	1 (33.3)	0 (0.0)	2 (66.7)	4 (66.7)	0 (0.0)	7 (33.3)
Stomach	0 (0.0)	0 (0.0)	0 (0.0)	0 (0.0)	0 (0.0)	0 (0.0)	0 (0.0)
Esophagus	0 (0.0)	0 (0.0)	0 (0.0)	0 (0.0)	0 (0.0)	1 (33.3)	1 (4.8)
Biliary tract	0 (0.0)	2 (66.7)	1 (33.3)	0 (0.0)	1 (16.7)	0 (0.0)	4 (19.0)
Liver	0 (0.0)	0 (0.0)	0 (0.0)	0 (0.0)	0 (0.0)	0 (0.0)	0 (0.0)
Others	1 (33.3)	0 (0.0)	1 (33.3)	0 (0.0)	1 (16.7)	1 (33.3)	4 (19.0)
Stage of cancer, *N* (%)							
IV	3 (100.0)	1 (33.3)	1 (33.3)	2 (66.7)	1 (16.7)	1 (33.3)	9 (42.9)
IVB	0 (0.0)	1 (33.3)	0 (0.0)	0 (0.0)	1 (16.7)	0 (0.0)	2 (9.5)
Refractory	0 (0.0)	1 (33.3)	2 (66.7)	1 (33.3)	4 (66.7)	2 (66.7)	10 (47.6)

	100 mg	200 mg	400 mg	600 mg	800 mg	1000 mg	Total
Patients (*N*)	3	3	3	3	6	3	21
Histopathological classification, *N* (%)							
Small round cell tumor							
	1 (33.3)	0 (0.0)	0 (0.0)	0 (0.0)	0 (0.0)	0 (0.0)	1 (4.8)
Invasive ductal carcinoma							
	0 (0.0)	0 (0.0)	0 (0.0)	1 (33.3)	0 (0.0)	0 (0.0)	1 (4.8)
Adenocarcinoma							
	2 (66.7)	3 (100.0)	2 (66.7)	2 (66.7)	5 (83.3)	1 (33.3)	15 (71.4)
Clear cell carcinoma							
	0 (0.0)	0 (0.0)	1 (33.3)	0 (0.0)	0 (0.0)	0 (0.0)	1 (4.8)
Squamous cell carcinoma							
	0 (0.0)	0 (0.0)	0 (0.0)	0 (0.0)	0 (0.0)	1 (33.3)	1 (4.8)
Acral lentiginous melanoma							
	0 (0.0)	0 (0.0)	0 (0.0)	0 (0.0)	0 (0.0)	1 (33.3)	1 (4.8)
Unknown							
	0 (0.0)	0 (0.0)	0 (0.0)	0 (0.0)	1 (16.7)	0 (0.0)	1 (4.8)
TNM staging (T) at study onset, *N* (%)							
T0	1 (33.3)	2 (66.7)	2 (66.7)	2 (66.7)	6 (100.0)	2 (66.7)	15 (71.4)
T3	1 (33.3)	1 (33.3)	1 (33.3)	0 (0.0)	0 (0.0)	0 (0.0)	3 (14.3)
T4	1 (33.3)	0 (0.0)	0 (0.0)	1 (33.3)	0 (0.0)	1 (33.3)	3 (14.3)

	100 mg	200 mg	400 mg	600 mg	800 mg	1000 mg	Total
Patients (*N*)	3	3	3	3	6	3	21
TNM staging (N) at study onset, *N* (%)							
N0	1 (33.3)	2 (66.7)	2 (66.7)	3 (100.0)	3 (50.0)	0 (0.0)	11 (52.4)
N1	2 (66.7)	1 (33.3)	1 (33.3)	0 (0.0)	1 (16.7)	1 (33.3)	6 (28.6)
N2	0 (0.0)	0 (0.0)	0 (0.0)	0 (0.0)	0 (0.0)	1 (33.3)	1 (4.8)
N2b	0 (0.0)	0 (0.0)	0 (0.0)	0 (0.0)	2 (33.3)	0 (0.0)	2 (9.5)
N3	0 (0.0)	0 (0.0)	0 (0.0)	0 (0.0)	0 (0.0)	1 (33.3)	1 (4.8)
TNM staging (M) at study onset, *N* (%)							
M0	0 (0.0)	0 (0.0)	0 (0.0)	0 (0.0)	1 (16.7)	0 (0.0)	1 (4.8)
M1	2 (66.7)	3 (100.0)	3 (100.0)	3 (100.0)	2 (33.3)	2 (66.7)	15 (71.4)
M1b	1 (33.3)	0 (0.0)	0 (0.0)	0 (0.0)	3 (50.0)	0 (0.0)	4 (19.0)
M1c	0 (0.0)	0 (0.0)	0 (0.0)	0 (0.0)	0 (0.0)	1 (33.3)	1 (4.8)
Treatment history: surgery*, *N* (%)							
0	2 (66.7)	1 (33.3)	1 (33.3)	0 (0.0)	0 (0.0)	1 (33.3)	5 (23.8)
≥1	1 (33.3)	2 (66.7)	2 (66.7)	3 (100.0)	6 (100.0)	2 (66.7)	16 (76.2)
Complete resection*							
	0 (0.0)	1 (33.3)	2 (66.7)	1 (33.3)	4 (66.7)	2 (66.7)	10 (47.6)
Residual tumor present*							
	0 (0.0)	1 (33.3)	0 (0.0)	2 (66.7)	3 (50.0)	1 (33.3)	7 (33.3)
Unknown*	1 (33.3)	0 (0.0)	0 (0.0)	0 (0.0)	2 (33.3)	0 (0.0)	3 (14.3)

	100 mg	200 mg	400 mg	600 mg	800 mg	1000 mg	Total
Patients (*N*)	3	3	3	3	6	3	21
Treatment history: radiotherapy, *N* (%)							
0	2 (66.7)	2 (66.7)	3 (100.0)	3 (100.0)	6 (100.0)	2 (66.7)	18 (85.7)
≥1	1 (33.3)	1 (33.3)	0 (0.0)	0 (0.0)	0 (0.0)	1 (33.3)	3 (14.3)
Prior systemic regimens, *N* (%)							
0	0 (0.0)	0 (0.0)	0 (0.0)	0 (0.0)	0 (0.0)	0 (0.0)	0 (0.0)
1	2 (66.7)	0 (0.0)	0 (0.0)	0 (0.0)	0 (0.0)	1 (33.3)	3 (14.3)
2	1 (33.3)	1 (33.3)	2 (66.7)	1 (33.3)	1 (16.7)	0 (0.0)	6 (28.6)
3	0 (0.0)	0 (0.0)	0 (0.0)	1 (33.3)	1 (16.7)	1 (33.3)	3 (14.3)
4	0 (0.0)	1 (33.3)	0 (0.0)	1 (33.3)	1 (16.7)	0 (0.0)	3 (14.3)
5	0 (0.0)	0 (0.0)	0 (0.0)	0 (0.0)	1 (16.7)	1 (33.3)	2 (9.5)
6	0 (0.0)	1 (33.3)	1 (33.3)	0 (0.0)	2 (33.3)	0 (0.0)	4 (19.0)
Comorbidities, *N* (%)							
0	0 (0.0)	0 (0.0)	0 (0.0)	0 (0.0)	0 (0.0)	0 (0.0)	0 (0.0)
≥1	3 (100.0)	3 (100.0)	3 (100.0)	3 (100.0)	6 (100.0)	3 (100.0)	21 (100.0)
Past medical history, *N* (%)							
0	3 (100.0)	3 (100.0)	2 (66.7)	3 (100.0)	3 (50.0)	1 (33.3)	15 (71.4)
≥1	0 (0.0)	0 (0.0)	1 (33.3)	0 (0.0)	3 (50.0)	2 (66.7)	6 (28.6)
KRAS mutations determined, *N* (%)							
	0 (0.0)	1 (33.3)	0 (0.0)	3 (100.0)	0 (0.0)	0 (0.0)	4 (19.0)

*Multiple answers were counted cumulatively.
